# The relationship of muscle oxygen saturation analyzer with other monitoring and quantification tools in a maximal incremental treadmill test

**DOI:** 10.3389/fphys.2023.1155037

**Published:** 2023-05-18

**Authors:** Florent Osmani, Carlos Lago-Fuentes, Josep Alemany-Iturriaga, Martín Barcala-Furelos

**Affiliations:** ^1^ Faculty of Health Sciences, Universidad Europea del Atlántico, Santander, Spain; ^2^ Faculty of Social Sciences and Humanities, Universidad Europea del Atlántico, Santander, Spain

**Keywords:** heart rate, lactate, NIRS, power meter, RPE

## Abstract

**Introduction:** The study aims to explore whether NIRS derived data can be used to identify the second ventilatory threshold (VT2) during a maximal incremental treadmill test in non-professional runners and to determine if there is a correlation between SmO_2_ and other valid and reliable exercise performance assessment measures or parameters for maximal incremental test, such as lactate concentration (LT), RPE, HR, and running power (W).

**Methods:** 24 participants were recruited for the study (5 women and 19 men). The devices used consisted of the following: i) a muscle oxygen saturation analyzer placed on the vastus lateralis of the right leg, ii) the Stryd power meter for running, iii) the Polar H7 heart rate band; and iv) the lactate analyzer. In addition, a subjective perceived exertion scale (RPE 1-10) was used. All of the previously mentioned devices were used in a maximal incremental treadmill test, which began at a speed of 8 km/h with a 1% slope and a speed increase of 1.2 km/h every 3 min. This was followed by a 30-s break to collect the lactate data between each 3-min stage. Spearman correlation was carried out and the level of significance was set at *p* < 0.05.

**Results:** The VT2 was observed at 87,41 ± 6,47% of the maximal aerobic speed (MAS) of each participant. No relationship between lactate data and SmO_2_ values (*p* = 0.076; *r* = −0.156) at the VT2 were found. No significant correlations were found between the SmO_2_ variables and the other variables (*p* > 0.05), but a high level of significance and strong correlations were found between all the following variables: power data (W), heart rate (HR), lactate concentration (LT) and RPE (*p* < 0.05; *r* > 0.5).

**Discussion:** SmO_2_ data alone were not enough to determine the VT2, and there were no significant correlations between SmO_2_ and the other studied variables during the maximal incremental treadmill test. Only 8 subjects had a breakpoint at the VT2 determined by lactate data.

**Conclusion:** The NIRS tool, Humon Hex, does not seem to be useful in determining VT2 and it does not correlate with the other variables in a maximal incremental treadmill test.

## 1 Highlights

- Humon Hex is a small, portable and non-invasive device for measuring local SmO_2_ in a muscle and it provides instant feedback.
- Humon Hex is not a good device for identifying VT2 during a maximal incremental treadmill test.
- The Humon Hex monitor showed no correlation between SmO_2_ and the other variables such as lactate, heart rate, RPE, and W during a maximal incremental treadmill test.

## 2 Introduction

Monitoring and quantification in sports is necessary in order to improve sports performance, such as the muscle oxygen saturation variable, which is of great interest to both improve performance and to prevent the risk of injury in athletes (Farzam et al., 2018). In endurance sports, exercise intensity in the maximal lactate steady state is generally considered the “gold standard” to assess the athlete’s aerobic and endurance capacity, in addition to being able to prescribe training based on this variable (Beneke, 2003). It is also a precise variable in the sports performance of endurance athletes (Haverty et al., 1988).

Physiological thresholds such as lactate thresholds or ventilatory thresholds can have a direct affect on endurance performance and are used to prescribe exercise intensity for training, competition, and different tests (Bentley et al., 2007). Lactate thresholds can be determined using different methods, such as fixed blood lactate concentration (FBLC) or onset of blood lactate accumulation (OBLA), which determine the first lactate threshold at 2 mmol/L and the second lactate threshold at 4 mmol/L, respectively (Bentley et al., 2007; Kindermann et al., 1979). Individual thresholds are determined using different formulas (6). All of these methods have high correlations between velocity and power output at the lactate threshold (Faude et al., 2009). Maximal aerobic speed (MAS) is defined as the minimum running speed necessary to attain maximal oxygen uptake (V̇O2 max) during an incremental treadmill running test (Billat et al., 1994). Another tool, such as the running power meter, estimates mechanical work and the characteristics of each running step and stride. These functions make it very useful for optimizing performance and preventing injuries (Luedke et al., 2016). The subjective Rating of Perceived Exertion (RPE) measurement is a subjective effort chart that starts at 1 and goes up to 10, with 1 being the lightest and 10 being the maximum effort. This scale is very useful because it is a simple, non-invasive and free method to monitor training load (Haddad et al., 2017). One last tool is the measurement of heart rate (HR), which is a popular method for monitoring cardiorespiratory and metabolic demands during running, since HR increases in a linear fashion with oxygen consumption and energy expenditure during the performance of incremental tests in the laboratory (Born et al., 2017). HR accuracy during physical exercise is important for monitoring and quantifying exercise intensity (Boudreaux et al., 2018).

Near-infrared spectroscopy (NIRS) is a validated tool for measuring venous oxygen saturation during exercise and rest (Mancini et al., 1985). It also has a high level of reliability during repeated incremental running and cycling tests (Austin et al., 2005). Despite the usefulness of this device in graded incremental tests, it is important to note that certain factors must be taken into account with this device, such as subcutaneous tissue, which includes muscle size, blood vessels, and adipose tissue. All these factors have an inference in the NIRS results, because the infrared rays measure is more difficult due to a thicker subcutaneous tissue. Additionally, there is currently a lack of standardization in the different using NIRS (Barstow, 1985). The lactate analyzer (Lactate Pro 2) was verified as reliable in research using a graded incremental rowing test, comparing it with a laboratory-based analyzer (Crotty et al., 2021). The same applied to the validity and reliability of the running power meter (Stryd) for variables of contact time and leg spring stiffness during a maximal incremental test (Imbach et al., 2020). Also, the study showed that it is a useful tool for quantifying running intensity at submaximal speeds (Imbach et al., 2020). The validity of the rating of perceived exertion (RPE) was verified in different sports. This verification study included a variety of sports disciplines, as cycling or running, and was found to be applicable in all scenarios, as graded or continuous intensity exercises (Haddad et al., 2017). In a separate study, the validity and reliability of the Polar H7 were evaluated using a graded cycling test and resistance exercises involving fifty subjects. The Polar H7 was compared to other HR monitoring devices, as Apple Watch Series, Fitbit or Garmin Vivosmart HR, and the results proved that it was not only the most valid and reliable device, but also the only device that was valid and reliable during all different levels of exercise intensity (Boudreaux et al., 2018).

Physiological thresholds such as lactate thresholds or ventilatory thresholds, are usually determined by incremental tests as a standard procedure (Bentley et al., 2007). But there is one main problem with maximal incremental tests to establish a lactate threshold, as there is a lack of consensus on the protocol to be used, i.e., in the variables of: total duration, duration of each stage, change in intensity of each stage, and total number of stages or incremental stages (Bentley et al., 2007; Jamnick et al., 2018). It was recommended that each stage last at least 3 min in order to be more reliable (Bentley et al., 2007). The number of stages during the test and the total duration of the test should depend on the initial intensity and power increments, although it is suggested that these increments be identical between each stage (Van Schuylenbergh et al., 2004).

In recent years, the measurement of muscle oxygen saturation (SmO_2_) has been increasing because it is based on NIRS technology, which is a portable and non-invasive tool that provides information on the change in oxygenation of the muscle tissue in which the tool is placed (Perrey and Ferrari, 2018). NIRS technology has been used to study diverse factors involved in sports, including biomechanics, the heterogeneity of the SmO_2_, muscle activation, muscle respiration, fatigue, and muscle damage caused by exercise. It has also been used to explore the effects of training and oxidative metabolism. This is in addition to being placed on different muscle groups such as the vastus lateralis, rectus femoris, gastrocnemius, deltoids, biceps, forearm, triceps, biceps femoris or vastus medialis. It was also used in different settings such as laboratory and field tests (Ferrari et al., 2011). SmO_2_ reflects the dynamic balance between oxygen supply and consumption (Ferrari et al., 2011). NIRS has been used in various training scenarios such as a long time trial, repeated sprints, a constant workload or in an incremental test (Perrey and Ferrari, 2018). During incremental exercises, the behavior of the SmO_2_ variable has four phases: i) an initial increase, ii) a linear or exponential decrease below the resting baseline values iii) leveling off when the participant reaches maximal volitional fatigue during this incremental test, being these results the lowest and below the resting baseline values and, and iv) an increase over baseline values during the first minutes of recovery (Bhambhani, 2004). There has been some controversy surrounding the use of NIRS devices during some incremental running or cycling tests, as some studies have shown correlations between ventilatory thresholds and SmO_2_ breakpoints (Austin et al., 2005; Feldmann et al., 2022), the first ventilatory threshold and SmO_2_ breakpoint (van der Zwaard et al., 2016), the second ventilatory threshold and SmO_2_ breakpoint (Fontana et al., 2015) or no clear correlations between ventilatory thresholds and SmO_2_ breakpoints (Racinais et al., 2014). For training monitoring purposes, NIRS is currently considered more useful than HR for monitoring running intensity in hilly terrain (Born et al., 2017).

The current researches aim to explore whether NIRS derived data can be used to identify the second ventilatory threshold (VT2) during a maximal incremental treadmill test in non-professional runners and to determine if there is a correlation between SmO_2_ and other valid and reliable exercise performance assessment measures or parameters for maximal incremental test, such as lactate concentration (LT), RPE, HR, and running power (W). The study will also investigate the relationship between SmO_2_ breakpoints and lactate thresholds. The maximal incremental treadmill test was chosen because it is useful in determining physiological thresholds for prescribing future exercise intensity for endurance sports. A 30-s break was included in the protocol to collect lactate data. This research seeks to address the lack of clarity in the scientific literature regarding the identification of the second ventilatory threshold during maximal incremental treadmill tests using a NIRS device and the correlation between NIRS-derived data and other monitoring tools.

## 3 Materials and methods

### 3.1 Study design

To answer the planned aim described above, a cross-sectional study design was carried out.

### 3.2 Participants

Initially, 24 participants were recruited for the study (5 women and 19 men). Three of the participants were excluded because the SmO_2_ measurements were erroneous. This was due to device movement, because in one of them it fell and the SmO_2_ measurements were not correct in the other two, possibly due to a limiting factor of this device. Finally, there were 21 participants (5 women and 16 men) who voluntarily participated in the study (mean ± standard deviation): age = 23.19 ± 4.18 years, weight = 68.93 ± 10.99 kg, and height = 1.75 ± 0.12 m. The inclusion criteria were: (I) performing a moderate exercise session at least 3 days a week for 30 min for the last 6 months, mainly involving movements such as running, cycling or swimming and (II) not having any injuries or disease which prevented the participant from performing the maximal incremental test. The exclusion criteria were: i) being in possession of an athletics sports license, which means that they are considered to be professional athletes. All participating subjects were informed of the protocol to be used and an informed consent form was signed before beginning participation in the study. The privacy of the data collected was respected following the principles of the Declaration of Helsinki. The study protocol was approved by the ethics committee of the European University of the Atlantic (CE-52).

### 3.3 Procedure

Firstly, the study procedure began with the recruitment of the convenience sample. All tests were performed in the University laboratory, at a temperature of ≈20°C. To start the test, all the measuring instruments were placed on the participants and they were checked to see that they worked correctly. The Humon Hex was placed on the vastus lateralis of the right leg, the Stryd was placed on the laces of the right shoe, the chest strap was adjusted with the Polar H7, and finally the RPE chart was explained. After that, a puncture was made in the lobe of the right ear in order to collect the lactate data later on. Before starting the maximal incremental test, different familiarization tests with the treadmill were explained and performed, so as to test the treadmill entry and exit protocol. The maximal incremental test began at a speed of 8 km/h, with a 1% slope and the speed was increased by 1.2 km/h every 3 min, and after each 3-min stage there was a 30-s stop to collect the blood sample (Campos et al., 2017). The test ended once 10 stages were completed or the participant reached their maximal volitional fatigue. The data was collected during the final phase of each step during the last 30 s. First of all, the running power was collected, followed by the HR, the % SmO_2_ and the RPE of the subject. The lactate data was collected during the 30-s stop phase and the test was always carried out under the same conditions, without receiving any feedback.

Once the participant finished the maximal test, recovery was performed for 3 min at a speed of 6 km/h, in which the HR and % SmO_2_ were recorded at the first minute and at the third minute. After recovery, the different devices were removed. As the participant left the laboratory, the data collected on the registration sheet was transferred to an Excel file to form the database. Finally, relative intensities were calculated by percentage of maximal aerobic speed (MAS), which was the last step completed.

### 3.4 Devices

A muscle oxygen saturation analyzer with NIRS technology (Humon Hex, Dynometrics Inc., Boston, MA, United States) was used to measure SmO_2_ in the vastus lateralis of the right leg. The lactate analyzer (Lactate Pro 2, Lactate Pro LT Arkay Inc., Kyoto, Japan) was used for lactate measurement with blood drops from the right ear lobe while the heart rate band (Polar H7, Polar Electro, Kemple, Finland) was used to measure the participant’s heart rate. The running power meter (Stryd Power meter, Stryd Inc., Boulder CO, United States) measured the running power of each step. In addition, the subjective perceived exertion scale (RPE 1-10) was used (Borg, 1998).

### 3.5 Statistical analysis

Descriptive data is presented as mean and standard deviation. The normality distribution of the data was verified through the Shapiro-Wilk test (*p* > 0.05), so the Spearman correlation test was carried out.

The criteria to interpret the magnitude of r for the previous correlation tests were the following: null correlation (0.00–0.09), smooth correlation (0.10–0.29), moderate correlation (0.30–0.49), good correlation (0.50–0.69), very good correlation (0.70–0.89), almost perfect correlation (0.90–0.99), and perfect correlation (1.00) (Hopkins et al., 2009).

Statistical significance was accepted at *p* < 0.05. Statistical analysis was performed with JASP (version 0.14.1.0, http://www.jasp-stats.org).

## 4 Results

The second ventilatory threshold (VT2) was observed (mean ± standard deviation): relative intensity = 87.41 ± 6.47% of MAS, speed = 12.62 ± 1.87 km/h, RPE = 6.58 ± 1.77 score, heart rate = 177.74 ± 12.63 beats per minute, watts = 252.79 ± 32.94 W and SmO_2_ = 60.47 ± 8.25.

Table 1 shows the maximal aerobic speed (MAS) values, the relative intensity of each step for each participant and their SmO_2_ values. The standard deviation of SmO_2_ goes from 4.04 to 11.12.

**TABLE 1:** Values of maximal aerobical speed, SmO2 and relative intensities for each participant.

Subject	MAS	RI_S1	SmO2_S1	RI_S2	SmO2_S2	RI_S3	SmO2_S3	RI_S4	SmO2_S4	RI_S5	SmO2_S5	RI_S6	SmO2_S6	RI_S7	SmO2_S7	RI_S8	SmO2_S8	RI_S9	SmO2_S9	RI_S10	SmO2_S10
1	16,4	48,78	58	56,1	58	63,41	60	70,73	61	78,05	57*	85,37	48	92,68	37	100	22				
2	14	57,14	23	65,71	45	74,29	58	82,86	58	91,43	55*	100	46								
3	11,6	68,97	50	79,31	60	89,66	71	100	68*												
4	14	57,14	66	65,71	68	74,29	69	82,86	69	91,43	68*	100	66								
5	17,6	45,45	58	52,27	58	59,09	64	65,91	70	72,73	69	79,55	67*	86,36	66	93,18	66	100	64		
7	11,6	68,97	50	79,31	52	89,66	55	100	54*												
8	14	57,14	67	65,71	69	74,29	72	82,86	72	91,43	70*	100	68								
9	15,2	52,63	65	60,53	68	68,42	71	76,32	71	84,21	70*	92,11	69	100	68						
10	14	57,14	66	65,71	65	74,29	59*	82,86	57	91,43	55	100	52								
11	14	57,14	64	65,71	65	74,29	68	82,86	70	91,43	70	100	69*								
12	18,8	42,55	48	48,94	60	55,32	59	61,7	63	68,09	65	74,47	64*	80,85	62	87,23	58	93,62	54	100	54
13	15,2	52,63	58	60,53	58	68,42	63	76,32	66	84,21	66	92,11	63*	100	59						
14	16,4	48,78	69	56,1	68	63,41	70	70,73	69*	78,05	68	85,37	67	92,68	66	100	64				
15	14	57,14	68	65,71	71	74,29	72	82,86	72	91,43	71*	100	68								
16	14	57,14	54	65,71	54	74,29	58	82,86	60	91,43	60	100	58*								
18	12,8	62,5	60	71,88	60	81,25	62	90,63	62	100	60*										
19	11,6	68,97	52	79,31	55	89,66	59	100	59												
21	14	57,14	56	65,71	53	74,29	55	82,86	54*	91,43	52	100	49								
22	14	57,14	64	65,71	65	74,29	65	82,86	64*	91,43	61	100	59								
23	14	57,14	58	65,71	64	74,29	65	82,86	63*	91,43	57	100	53								
24	14	57,14	62	65,71	64	74,29	66	82,86	66	91,43	63*	100	59								

MAS (km/h), maximal aerobical speed; RI (% of MAS): relative intensities of MAS; S1: step 1; SmO2 (%): Saturation of muscle oxygen. * = point of inflection of estimated VT2 by SmO2.

Table 2 shows the MAS values, the relative intensity of each step for each participant and their lactate concentration values (LT). Most of the participants have their second lactate threshold between stage 4 and 5, and these stages have a speed of 11.6 km/h and 12.8 km/h.

**TABLE 2:** Values of maximal aerobical speed, lactate concentration and relative intensities for each participant.

Subject	MAS	RI_S1	LT_S1	RI_S2	LT_S2	RI_S3	LT_S3	RI_S4	LT_S4	RI_S5	LT_S5	RI_S6	LT_S6	RI_S7	LT_S7	RI_S8	LT_S8	RI_S9	LT_S9	RI_S10	LT_S10
1	16,4	48,78	1,6	56,1	1,5	63,41	1,7	70,73	2,1	78,05	2,3	85,37	3,4	92,68	4,1*	100	6,3				
2	14	57,14	1,5	65,71	1,6	74,29	1,8	82,86	3,5	91,43	5*	100	6,8								
3	11,6	68,97	1,4	79,31	1,6	89,66	2,5	100	5*												
4	14	57,14	2,5	65,71	3	74,29	3,5	82,86	4,2*	91,43	4,4	100	7,8								
5	17,6	45,45	1,3	52,27	1,2	59,09	0,9	65,91	1,1	72,73	1,8	79,55	2,5	86,36	4*	93,18	4,2	100	5		
7	11,6	68,97	2,7	79,31	3,7	89,66	5,5*	100	7,8												
8	14	57,14	2,1	65,71	2,8	74,29	3,1	82,86	3,9	91,43	6,5*	100	10,2								
9	15,2	52,63	1,9	60,53	1,7	68,42	2,8	76,32	3,3	84,21	5,2*	92,11	5,9	100	9,6						
10	14	57,14	1,9	65,71	2,7	74,29	1,9	82,86	2,6	91,43	3,8	100	5,2*								
11	14	57,14	1,5	65,71	2,5	74,29	1,9	82,86	2,2	91,43	3,7	100	3,9								
12	18,8	42,55	2,1	48,94	2,3	55,32	1,7	61,7	2	68,09	2,5	74,47	3	80,85	3,9	87,23	4,5*	93,62	7,4	100	10,5
13	15,2	52,63	1,7	60,53	2,1	68,42	2,7	76,32	2,8	84,21	3	92,11	5,7*	100	7						
14	16,4	48,78	1,4	56,1	1,2	63,41	1,2	70,73	1,8	78,05	2,1	85,37	2,7	92,68	3,7	100	4,5*				
15	14	57,14	1,6	65,71	1,9	74,29	1,5	82,86	1,9	91,43	2,2	100	2,9								
16	14	57,14	1,4	65,71	2	74,29	2,4	82,86	2,9	91,43	4,4*	100	5,5								
18	12,8	62,5	1,6	71,88	1,8	81,25	1,8	90,63	3	100	5,6*										
19	11,6	68,97	3,1	79,31	4,9*	89,66	7,5	100	11,1												
21	14	57,14	1,7	65,71	2,2	74,29	3,1	82,86	4*	91,43	5,4	100	7,8								
22	14	57,14	1,4	65,71	2,4	74,29	2,7	82,86	3,1	91,43	4,2*	100	6								
23	14	57,14	1,5	65,71	1,5	74,29	1,8	82,86	2,1	91,43	2,9	100	4,4*								
24	14	57,14	1,3	65,71	1,7	74,29	2	82,86	2,9	91,43	4,2*	100	5,7								

MAS (km/h): maximal aerobical speed; RI (% of MAS): relative intensities of MAS; S1: step 1; LT (mmol/L): Lactate concentration. * = Anaerobic threshold by 4 mmol/L of LT.

No significant correlations were found between the variables of SmO_2_ and HR (*r* = −0.028; *p* = 0.752), SmO_2_ and W (*r* = 0.047; *p* = 0.593) and SmO_2_ and RPE (*r* = 0.035; *p* = 0.692), as these were results from null correlations, while the correlation between SmO_2_ and lactate (*r* = −0.118; *p* = 0.177) is slightly negative, but without significance. These correlations can be seen in Table 3.

**TABLE 3:** Correlations and significance of the used variables for the measurement in the maximal incremental test on treadmill.

Performance variables			Spearman’s rho		*p*
HR	—	**SMO2**	−0.028		0.752
HR	—	**W**	0.598	***	<.001
HR	—	**LT**	0.717	***	<.001
HR	—	**RPE**	0.776	***	<.001
SMO2	—	**W**	0.047		0.593
SMO2	—	**LT**	−0.118		0.177
SMO2	—	**RPE**	0.035		0.692
W	—	**LT**	0.716	***	<.001
W	—	**RPE**	0.808	***	<.001
LT	—	**RPE**	0.769	***	<.001

HR, heart rate; SMO2, percentage of muscle oxygen saturation; W, running power; LT, lactate concentration; RPE, range of perceived exertion.*p < .05, **p < .01, ***p < .001.

**FIGURE 1:**
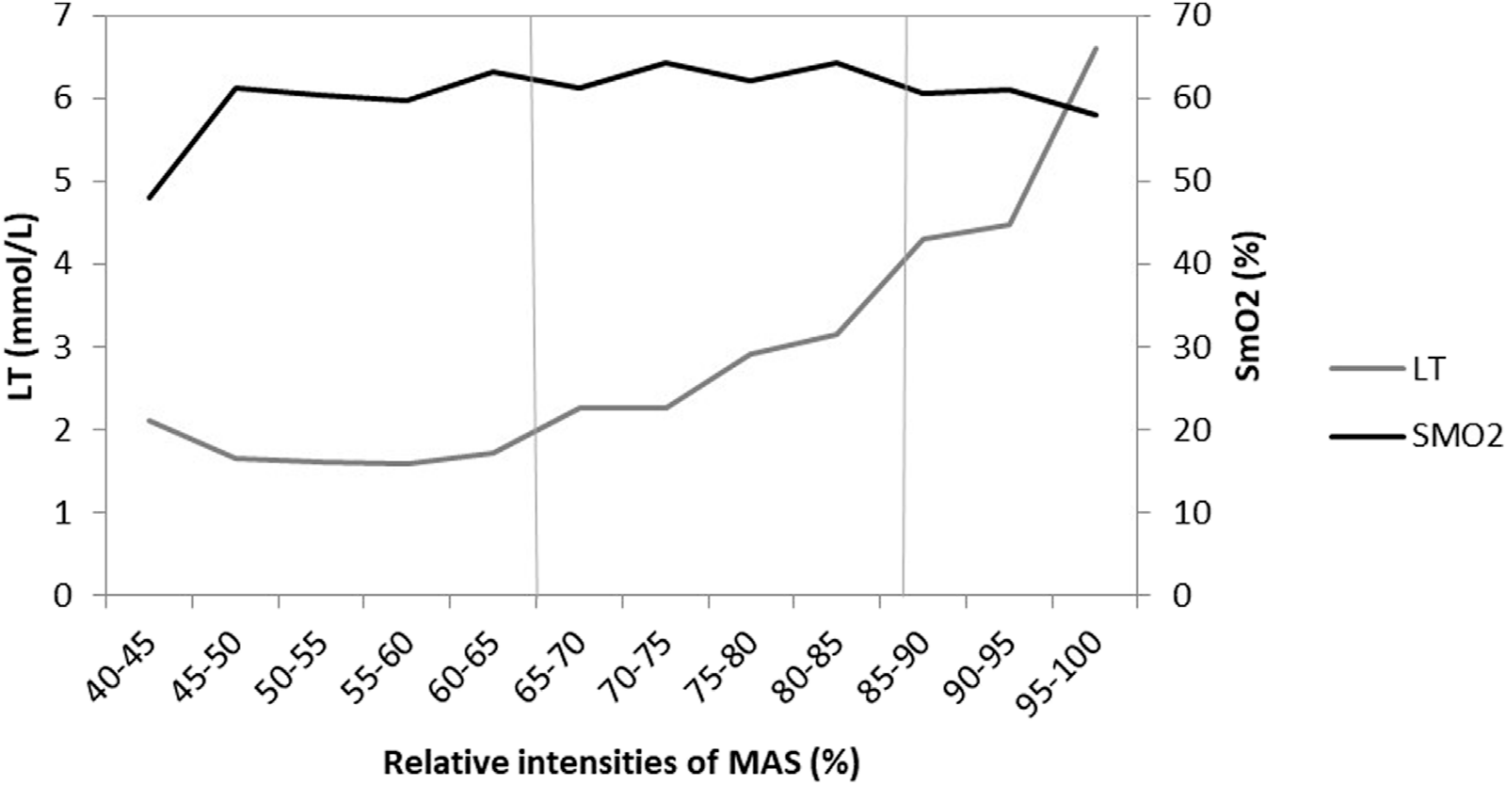
Relationship between lactate concentration and muscle oxygen saturation at each MAS relative intensity of each participant. SmO2—The percentage of muscle oxygen saturation. LT—Lactate concentration in mmol/L. MAS: Maximal aerobic speed.

In the comparison between the results of lactate and SmO_2_ in Figure 1 (*p* = 0.076; *r* = −0.156), we can see that there is no inflection point in the lactate thresholds with fixed concentrations (2 mmol/L and 4 mmol/L) and SmO_2_. The VT2 is at 85%–90% of MAS of each participant, being the mean value 4,3 mmol/L, but SmO_2_ does not show an inflection point at these relative intensities. Also, when verifying individual data, it can be seen that only 8 of them have some inflection point at SmO_2_ values at the same point as the VT2, 10 of them have no inflection point and 3 participants did not reach 4 mmol/L to determine the VT2.

Nevertheless, in the results of the Spearman correlation (Table 3), we can observe a great significance between all the variables of W, HR, LT, and RPE, with good correlations, *r* = (0.5–0.69), or very good, *r* = (0.7–0.89).

**FIGURE 2:**
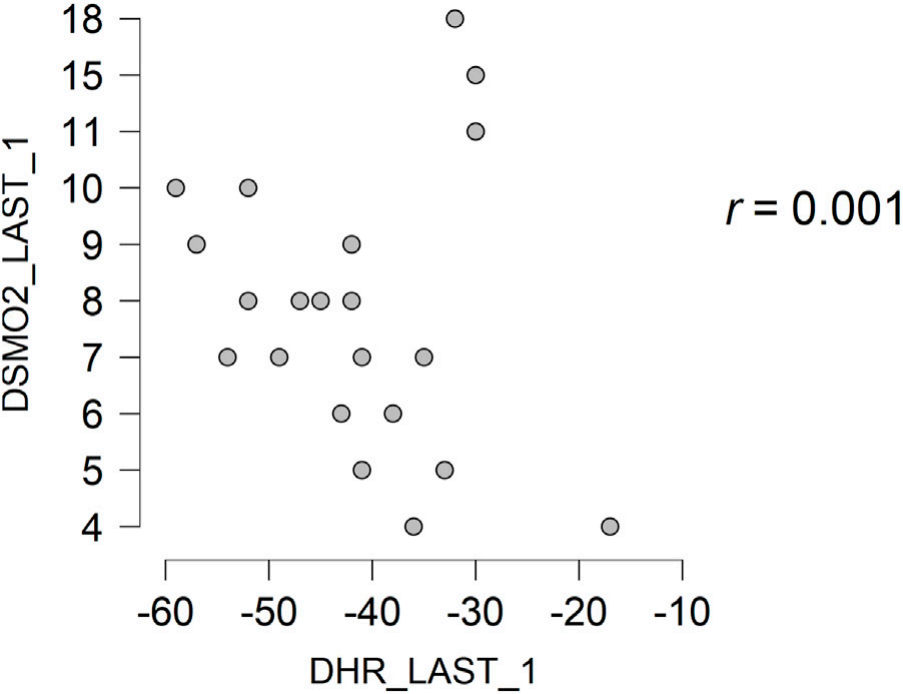
Correlation between the variables of SmO2 and HR from the last completed step to the first minute of recovery and from the first minute of recovery to the 3 min of recovery.

**FIGURE 3:**
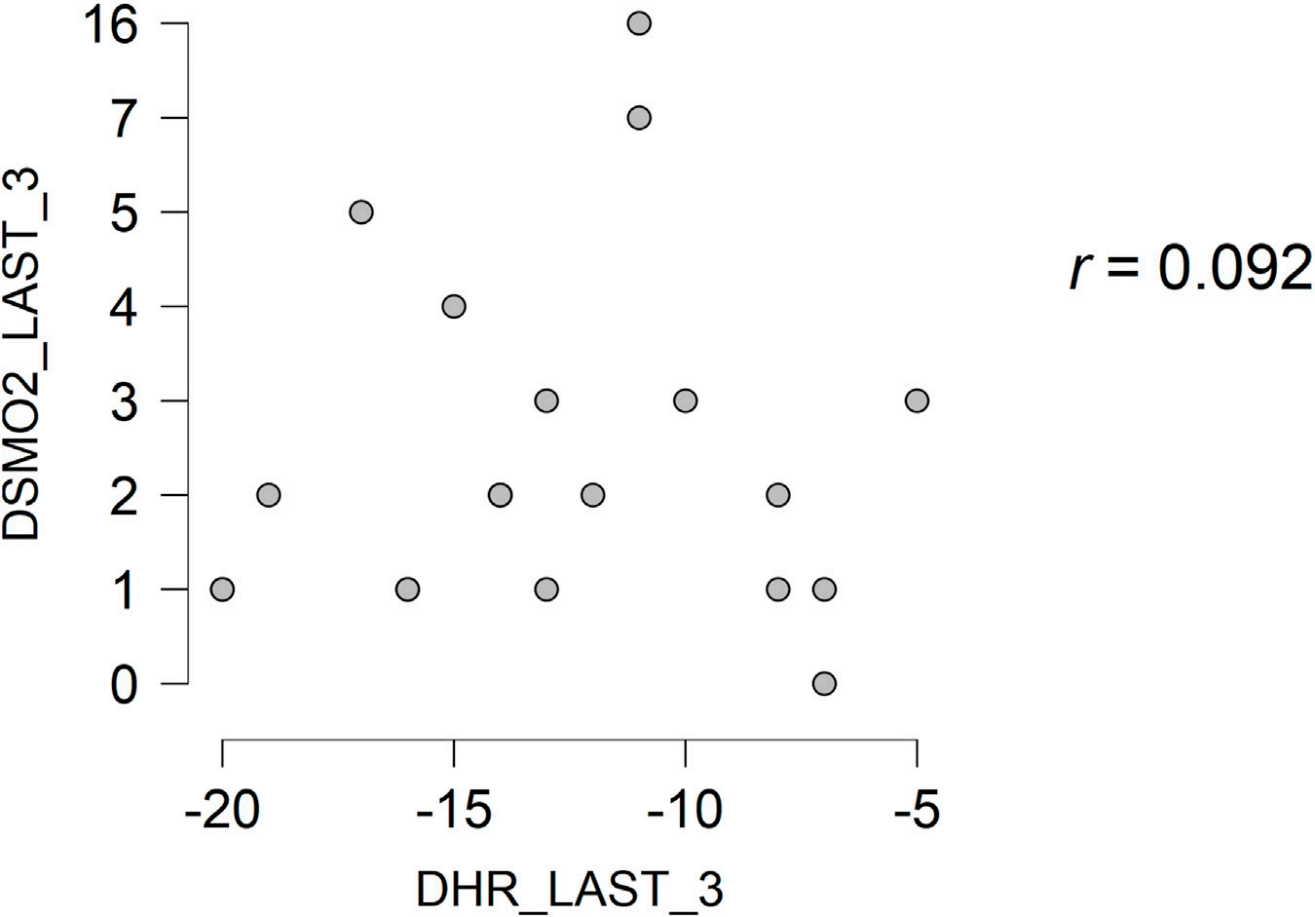
Correlation between the variables of SmO2 and HR from the first minute of recovery to the 3 min of recovery. SmO2—The percentage of muscle oxygen saturation. HR—Heart Rate. DSMO2_LAST_1—The difference between the percentage of muscle oxygen saturation since the last completed step to the first recovery minute. DHR_LAST-1—The difference between the heart rate since the last completed step to the first recovery minute. DSMO2_LAST_3—The difference between the percentage of muscle oxygen saturation since the last completed step to the third minute of recovery. DHR_LAST_3—The difference between the heart rate since the last completed step to the 3 min of recovery.

Regarding the recovery of the participants, it is observed that there is a null and non-significant correlation between the variables of SmO_2_ and HR, from the last step completed and the first minute of recovery (*r* = 0.001; *p* = 0.997) (Figure 2), and from the first minute of recovery up to 3 min of recovery (*r* = 0.092; *p* = 0.690) (Figure 3).

## 5 Discussion

The main objective of the study was to identify the second ventilatory threshold during incremental tests using NIRS-derived data and to find out if these data correlated with other variables through a maximal incremental treadmill test in non-professional runners.

We found that the SmO_2_ data alone were not enough to determine the VT2, and there were no significant correlations between SmO_2_ and the other studied variables during the maximal incremental treadmill test. Only 8 subjects had a breakpoint at the VT2 determined by lactate data, so SmO_2_ is not sensitive enough to determine the VT2 by itself. This result is consistent with a previous study using a maximal cycle-ergometer ramp test with 25 cyclists (Racinais et al., 2014). However, other studies using incremental tests in cycling or running have observed correlations between one or both ventilatory thresholds and SmO_2_ (Austin et al., 2005; Fontana et al., 2015; van der Zwaard et al., 2016; Feldmann et al., 2022), which may be due to different NIRS devices used or certain differences in the protocol used, as graded incremental test with a lower speed increase (0.8 km/h each 3 min) than in our study (Austin et al., 2005) or a 30 s stages test with speed increase of 0.5 km/h until to exhaustion (Feldmann et al., 2022). In addition to this, it should be noted that in our study, a 30-s break was used between stages during the test. This factor may have exerted an impact on the outcomes, given that the 30-s break may have allowed for sufficient muscle recovery, thus affecting the determination of the ventilatory thresholds. We also found that SmO_2_ had significant variability between subjects, which could be a result of the limitations of the NIRS tool due to subcutaneous tissue (Barstow, 1985), but we cannot confirm it as this has not been measured. According to other studies (Thiel et al., 2011; Crum et al., 2017), it is shown that at higher intensity there is a greater standard deviation of SmO_2_, obtaining high values ​​of standard deviation during the entire test on all the different relative intensities. This corroborates the great individual variability that this variable has, as already studied in another incremental test protocol. This protocol involved a race with 46 participants, of which 25 were runners and 21 were cyclists, all of whom were professional athletes who trained between 5 and 7 days a week (Austin et al., 2005). SmO_2_ seems to be a good indicator of effort for variable efforts, which are exercises with a changing intensity, such as on uphill and downhill terrain (Born et al., 2017), while it does not seem to be a great indicator of effort for incremental efforts (Paredes-Ruiz et al., 2020). However, there is some discrepancy since it does state that it is actually valid in an incremental cycle machine test (Crum et al., 2017), and in another incremental race test it is mentioned that it depends on the subject (Austin et al., 2005). Also, Humon Hex seems to be a good device to measure SmO_2_ in the vastus lateralis in endurance runners for submaximal 60 min run-to-exhaustion treadmill running test (Jaén-Carrillo et al., 2022). In this study, the SmO_2_ variable was not observed as a useful tool of effort and to determine the VT2 for a maximal incremental test in treadmill running, due to its great inter-subject variability and low intra-subject variability, without any clear inflection point for VT2. In addition to the above, in the designed incremental protocol, 3 participants were discarded due to erroneous measurements. These errors may haven been caused by various factors such as: i) the swinging motion generated by running, ii) changes in muscle contractions during the race, iii) the impact of running, and iv) sweat. All of these limiting factors have the potential to influence the accuracy of the NIRS device measurements and the correct placement of the sensor during running tests.

Lactate is the variable which is considered the “gold standard” for assessing aerobic capacity in endurance sports (Beneke, 2003), with HR being the most popular and simplest method to monitor and quantify on a daily basis (Born et al., 2017). Therefore, both variables are used in an essential way for incremental tests, and the gas analyzer is the other main tool in these tests. Therefore, Figure 1 shows the relationship of SmO_2_ and lactate during the test, which does not show any inflection point to determine lactate thresholds of SmO_2_ data. Although SmO_2_ has a little decrease at 85%–90% RI, it does not continue decreasing. So, it is not possible to determine a VT2 by SmO_2_ values. It should be noted that, according to the fixed lactate concentration model, ventilatory thresholds are typically located at lactate thresholds ranging from 2 to 4 mmol/L (Kindermann et al., 1979; Heck et al., 1985; Corrales-Gil et al., 2013). However, there is significant controversy regarding the different methods used to determine lactate thresholds, with many being deemed valid for this purpose. Given this, the present study chose to utilize the fixed lactate concentration model as it is a valid method for determining physiological thresholds (Faude et al., 2009). In addition, the correlation between lactate and SmO_2_ is mildly negative and without significance. Therefore, these results suggest that Humon Hex is not useful for determining lactate thresholds, as has already been observed in other studies that determined that there is no correlation between lactate and SmO_2_, nor can thresholds be estimated (Austin et al., 2005; Perrey and Ferrari, 2018). A possible cause of this could be the great variability between subjects (Anderson, 2017). This is due to the fact that no absolute value was found for the different lactate thresholds, in addition to the fact that no negative exponential difference was found for SmO_2_, which was in accordance with the exponential growth of lactate. Some studies had good results to determine ventilatory thresholds during graded exercise test with a bicycle ergometer; these results could differ with this study due to the different type of exercise and a different maximal incremental test, because in our study it has a 30 s stop between steps, which could change the results (Lin et al., 2020; Salas-Montoro et al., 2022). Also note that the NIRS device only measures a muscle locally, whereas lactate is collected directly from earlobe blood droplets.

However, SmO_2_ does not have a correlation with HR. This was observed in other studies in which runners were analyzed on changing terrain, with ascents and descents (Born et al., 2017), and although it does not have a correlation, it does prove to be a complementary tool to the HR for this type of situation. Furthermore, SmO_2_ could be a better monitoring variable for hilly terrain than HR, but further research is necessary on this topic. This is explained by the fact that both variables are affected by different external factors, such as muscle contractions or thermoregulation/dehydration (Perrey and Ferrari, 2018). In addition, SmO_2_ also did not present correlations with variables such as power (W) and RPE, as these variables were previously validated for maximal incremental efforts (Haddad et al., 2017; Imbach et al., 2020). Therefore, our findings seem to indicate that SmO_2_ is not a useful variable for a maximal incremental treadmill test with 30 s stop phase, as no significant correlation was found with all the other validated variables, in addition to several limitations such as running oscillation or the measurement of only a local muscle as a measure of internal load, but this can differ depending on the type of NIRS device. Nevertheless, regarding the correlated variables between lactate, HR, RPE and W, different positive correlations were observed, from good to very good and with great significance (*r* > 0.6, *p* < 0.05). These findings have already been found in many other studies (Montagu, 1962; Paton and Hopkins, 2001; Van Schuylenbergh et al., 2004; Corrales-Gil et al., 2013; Abe et al., 2015; Haddad et al., 2017; García-Pinillos et al., 2019).

Regarding the recovery relationship between SmO_2_ and HR, both in the first minute and in the first 3 min, the correlations observed were almost null (*r* < 0.1: *p* > 0.05). It was taken into account that HR is a good variable to determine recovery, in addition to having a good correlation with lactate to measure recovery. However, this was studied in research involving only 2 professional middle-distance runners, in which a decrease in lactate was accompanied by HR (Campos et al., 2017). With these results, it is not recommended to use the Humon Hex as a tool to measure recovery after a maximal incremental test on a treadmill, since the Humon data does not accompany the decrease in HR with an increase in muscle oxygen saturation in any significant way.

Despite the findings obtained, it is necessary to highlight certain limitations of this research. Firstly, the sample size was low compared to other investigations (n = 21). However, given the inclusion and exclusion criteria along with the need to use physiological variables, our findings are relevant to the scientific and sports community. In addition, 5 people in the sample were women, which is a good starting point for carrying out this same study solely with a female population in the future, given the different behavior of the physiological variables in submaximal efforts. Lastly, it was not possible to measure the subcutaneous tissue in order to take it into account in the evaluation of the results.

For future research studies, use of the Humon Hex device is highly recommended for variable intensity or changing terrain activities, where it has been shown to have greater usefulness and validity in its measurements (Born et al., 2017). It is also suggested to consider using this tool in sports that involve less oscillation, such as cycling, which is a more stationary sport and does not have the impact that could affect SmO_2_ measurements. Furthermore, it is essential to study how the stop phases during a maximal incremental test could affect the behavior of SmO_2_ in determining threshold breakpoints.

## 6 Conclusion

The study aimed to explore whether NIRS-derived data could be used to identify the second ventilatory threshold (VT2) during a maximal incremental treadmill test involving non-professional runners and to determine if there was a correlation between SmO_2_ and other exercise performance assessment measures or parameters, such as lactate concentration (LT), RPE, HR, and running power (W). The conclusion of the study is that the NIRS tool, Humon Hex, does not seem to be useful for determining VT2 in a maximal incremental treadmill test. Also, SmO_2_ values of this device do not correlate with other variables in these types of tests (*p* > 0.05). Furthermore, SmO_2_ does not seem a good indicator for measuring recovery versus using HR.

## Data Availability

The raw data supporting the conclusion of this article will be made available by the authors, without undue reservation.
